# iCatcher: A neural network approach for automated coding of young children's eye movements

**DOI:** 10.1111/infa.12468

**Published:** 2022-04-13

**Authors:** Yotam Erel, Christine E. Potter, Sagi Jaffe‐Dax, Casey Lew‐Williams, Amit H. Bermano

**Affiliations:** ^1^ School of Computer Science Tel Aviv University Tel Aviv Israel; ^2^ Department of Psychology Princeton University Princeton New Jersey USA; ^3^ Department of Psychology The University of Texas at El Paso El Paso Texas USA; ^4^ School of Psychological Sciences and Segol School for Neuroscience Tel Aviv University Tel Aviv Israel

## Abstract

Infants' looking behaviors are often used for measuring attention, real‐time processing, and learning—often using low‐resolution videos. Despite the ubiquity of gaze‐related methods in developmental science, current analysis techniques usually involve laborious post hoc coding, imprecise real‐time coding, or expensive eye trackers that may increase data loss and require a calibration phase. As an alternative, we propose using computer vision methods to perform automatic gaze estimation from low‐resolution videos. At the core of our approach is a neural network that classifies gaze directions in real time. We compared our method, called iCatcher, to manually annotated videos from a prior study in which infants looked at one of two pictures on a screen. We demonstrated that the accuracy of iCatcher approximates that of human annotators and that it replicates the prior study's results. Our method is publicly available as an open‐source repository at https://github.com/yoterel/iCatcher.

## 1 INTRODUCTION

Measurement of eye movements is one of the most widely used methods in developmental science. Infants, children, and adults alike tend to shift their eyes to relevant or interesting stimuli, and researchers have been trying to harness this behavior in careful ways for decades (Aslin, 2007; Eberhard et al., 1995; Fernald et al., 2008; Golinkoff et al., 1987; Lew‐Williams & Fernald, 2007; Trueswell, 2008). For example, in the 1950s, Professor Eleanor Maccoby hid behind a movie screen with two small holes, held a “timing button” in each of her hands, and aggregated the amount of time that participants looked left versus right (Maccoby, 2019).

Comparisons of looking toward one location versus another have been used extensively in research on infancy, for example, in studies of early language processing. With low task demands and high temporal precision, studies have used the looking‐while‐listening (LWL) procedure, also called the preferential looking procedure, to understand the emergence of infants' language comprehension, developmental changes in processing efficiency, and young children's sensitivity to various types of linguistic information (Bergelson & Swingley, 2012; Fernald et al., 1998; Trueswell & Gleitman, 2004). Because of the simplicity of the task, the LWL procedure can be used with a wide range of participants, including individuals of different ages and abilities, and can even be ported outside of the lab setting (Naigles & Tovar, 2012; Venker et al., 2020). Measures of looking toward a stimulus on one side of a screen versus the other have also been used to study diverse topics, including infants' object recognition (Fagan III, 1974), visual working memory (Ross‐Sheehy et al., 2003), and even social attention (Kinzler et al., 2007).

While the demands imposed upon the participant are minimal (e.g., looking at pictures and listening to speech), the demands experienced by the research team are less trivial—both for automatic eye trackers and manual coding methods. Importantly, each method introduces obstacles to broadening participation in developmental research in underrepresented regions in the world.

Automatic eye trackers (e.g., those manufactured by Tobii and EyeLink) offer high spatial and temporal resolution relative to other approaches (see Gredebäck et al., 2009 for a review of the uses of eye tracking in research on infancy). They allow for studies that measure looking at many different locations on a screen, and produce data quickly and without bias, making them powerful tools for research. However, studies where the measured behavior involves only looking toward one side of the screen versus the other do not require high spatial resolution. Hence, they do not justify the use of eye trackers, which typically cost tens of thousands of dollars. In addition, they impose specific procedural constraints. First, most automatic eye trackers require participants to remain in a relatively fixed position, which can be difficult for young children to maintain. Second, eye trackers are often designed to be compatible with a limited range of both hardware and software, reducing researchers' flexibility in designing experiments and processing data. Third, a calibration procedure must be carried out at the beginning of the study, which can vary in its reliability and prolong the length time that a young child must remain attentive (Aslin, 2012; Oakes, 2010). Perhaps most problematic for infant research, automatic eye trackers are prone to increased loss of data. Given the small number of trials and limited attention spans of infants and young children, this loss can decrease the reliability of results (Wass et al., 2014). Moreover, to use eye trackers, participants must come into a dedicated lab space, which may not be feasible for participants in many regions. The alternative—manual coding—is easier and more accessible in some ways, because a basic study can be implemented cheaply using a laptop with a video camera. With thorough training, research assistants typically achieve high inter‐rater reliability and high‐quality data, with relatively little data loss (Fernald et al., 2008). However, the coding process is labor intensive: a 5‐minute session usually takes 45–75 min to code, and achieving high intercoder reliability requires great vigilance. Venker et al. (2020) provide a detailed discussion on many benefits and disadvantages of automated and hand‐coded methods.

Other approaches have been proposed for enabling efficient evaluation of infant gaze. Aided by increasing computational power and the availability of large datasets, advances in deep learning techniques have revolutionized the capabilities of computer vision. Appearance‐based data‐driven approaches using neural networks have shown great success in various detection tasks from image datasets (Krizhevsky et al., 2020; Russakovsky et al., 2015), as well as head pose estimation and eye tracking (Wood et al., 2015; Zhang et al., 2015), with saltatory gains in recent years (Fischer et al., 2018). These methods overcome most of the limitations posed by physical eye‐tracking devices in that they do not require manual labor and they usually involve minimal loss of accuracy. They can also be deployed in various in‐the‐wild setups that are not confined to laboratories, enabling large‐scale remote experiments (Chouinard et al., 2019; Krafka et al., 2016). They do, however, require an extremely large collection of images that are sampled from the distribution of the relevant domain for training. In the case of eye tracking, this would require a collection of relevant images of participant faces, preferably in annotated format. Many other appearance‐based methods also exist, most of which rely on a calibration step performed by the user to maintain good accuracy and to compensate for not being data driven (WebGazer; Papoutsaki et al., 2016; Opengazer; Zieliński, 2020), making them much less useful for developmental or clinical populations. Moreover, these options are not sufficiently robust to contend with changes in the scene and subject appearance relative to the dominant deep‐learning‐based options. For example, when lighting conditions change or when there are participants of different ages and/or races, accuracy sometimes declines.

In the coming decades, gaze‐related methods will remain a staple in developmental science. How can we maximize the usefulness of this approach and make gaze‐related paradigms as accessible as possible to any research team from anywhere? To address the weaknesses inherent in hand coding and to avoid the limitations of a specialized eye tracker, we followed the recent trend of appearance‐based data‐driven approaches. We used a collection of annotated videos from previous studies to construct a solution—a program called iCatcher—that automatically annotates videos in real time, with accuracy that approximates that of human annotators. We showed that our openly available program faithfully replicates results from previous research that used manual coding, and also outperformed prior solutions, including automated eye tracking (e.g., Chouinard et al., 2019; Fischer et al., 2018), in the task of classifying frames into discrete categories (i.e., left vs. right, and left vs. right vs. away).

## 2 METHOD

To train iCatcher, we collected 266 video sessions of infants and young children (ages 18–72 months) who participated in different studies using the looking‐while‐listening procedure. The present study was conducted according to guidelines laid down in the Declaration of Helsinki, with written informed consent obtained from a parent or guardian for each child before any assessment or data collection. All procedures involving human subjects in this study were approved by the Princeton University IRB. Infants were seated on their parents' laps approximately 70 cm from a single 55″ screen and were recorded as they viewed pairs of images (sized 38 cm × 35 cm, with 43 cm between them) and heard sentences labeling one object. Illumination varied significantly between videos, and the background was mostly black (but not always). The videos were of relatively low resolution, and infants' faces usually consisted of approximately 80 × 80 pixels. The relatively dim and variable lighting and the low facial resolution were the main training‐related challenges in this dataset. Prior to creating iCatcher, trained research assistants had manually labeled each frame (at 33 ms intervals) of these videos for whether the infant was looking at the left or right image, or neither (“away”).

The training phase of iCatcher was performed once on a subset of the entire data (“the training set”) consisting of ∼80% of the videos. During training, we validated our model's performance on another disjoint subset of the data (“the validation set”) consisting of ∼10% of the videos. The other phase, the inference phase, allowed us to use the trained model to code videos in real time. We used the trained model and tested it on the remaining ∼10% of previously unseen videos (“test set”), which was the complete set of data from a recent study (Potter & Lew‐Williams, 2022).

### 2.1 Data set preparation

**FIGURE 1:**
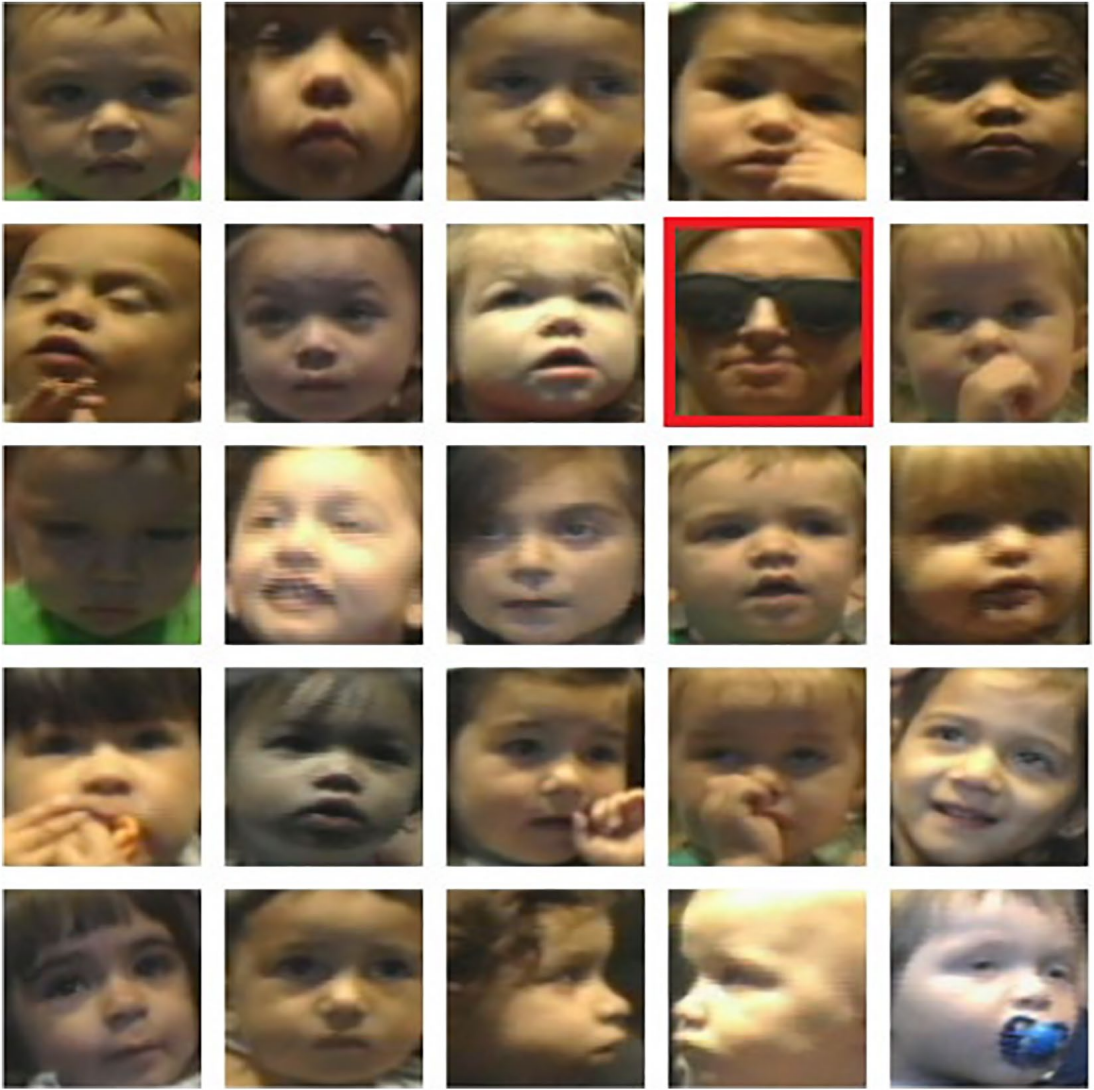
Samples of faces extracted from the video sessions using the face extractor (Bradski, 2000). *Note*. The red box highlights a failure case in which the face detector extracted the parent's face rather than the child's face. These crops were manually removed prior to training. Note that other face extractor programs can be used; we selected this face extractor program because of its ease of use and ability to recognize faces in various lighting conditions and with a relatively large range of face orientations

Our first step was to crop each infant's face from every frame using an off‐the‐shelf deep neural network‐based face extractor (Bradski, 2000), depicted in Figure 1. Note that extra care must be taken to filtering out faces of parents using this process. This can be done automatically using an age or id detection algorithm (see Cao et al., 2021 for an example), or through rapid manual inspection (taking roughly 2 h for the entire dataset). All cropped regions were resized to 75 × 75 pixels. Using a rolling window of 10 frames, we selected alternating frames (i.e., every odd element of every 10‐frame sequence) and treated them as a single data point. Each such data point was labeled with one of three classes (“left,” “right,” or “away”) representing the gaze state of the infant in the middle frame (the third frame out of five) as annotated by human coders. The entire dataset consisted of approximately 500,000 such data points.

### 2.2 Network architecture and training

**FIGURE 2:**
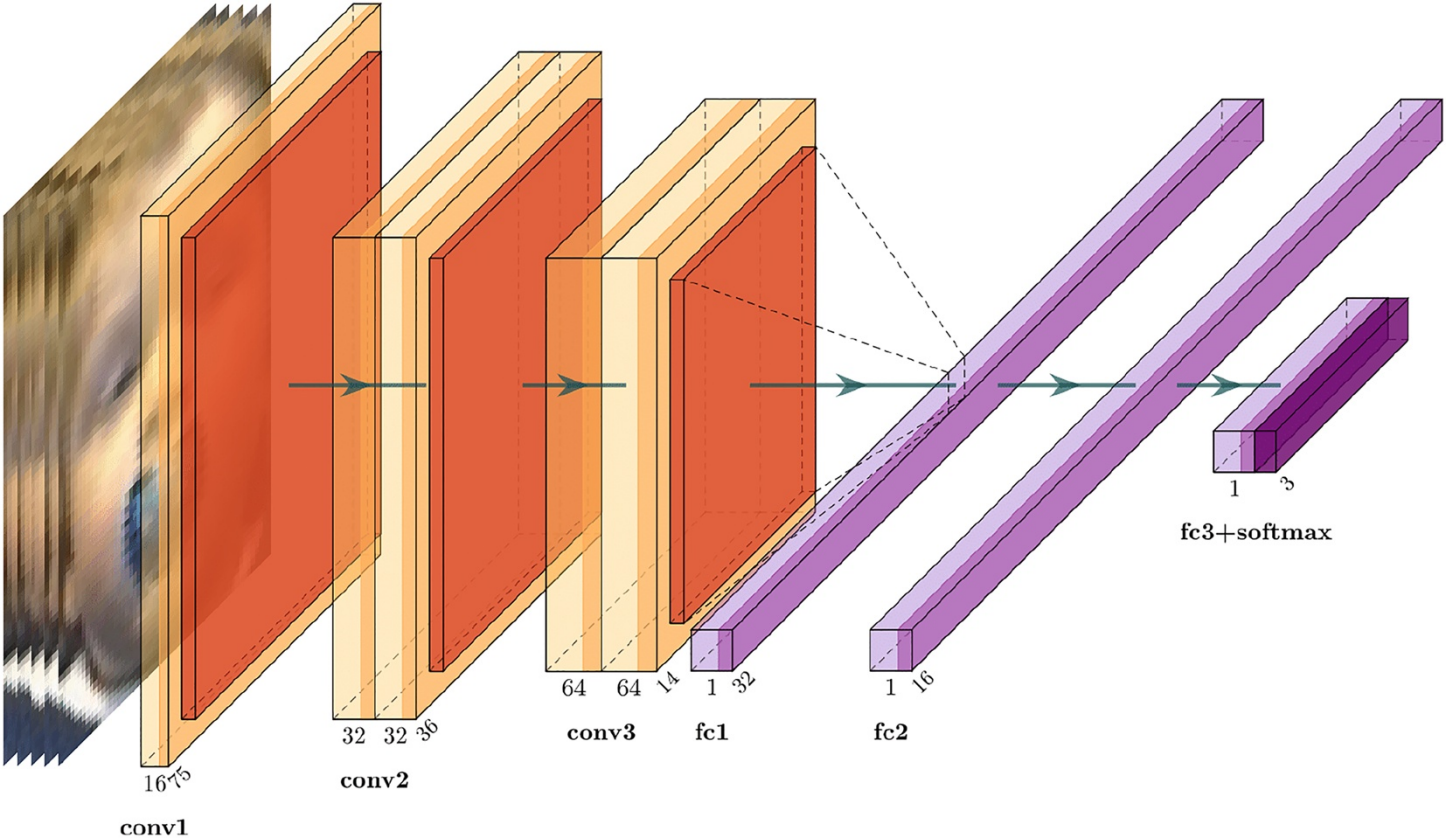
The network architecture of iCatcher. *Note*. First, cropped versions of input faces are taken from alternating consecutive frames and are resized to 75 × 75 pixels. Then, they are stacked in groups of five and fed to the network along with their annotated label. Labels beneath the convolutional blocks indicate the filter dimensions used. All convolutional layers used a kernel size of (3,3) with stride 1, and no padding. We used Max‐Pooling to pool after each convolutional block

When manually coding eye movements, human annotators label each individual frame in a video, and they usually do so by scanning the frames before and after the target frame to decide if a change has occurred. Exploiting this temporal context before is crucial to their ability to judge if the eyes have begun moving toward a new location. This process is often part of instruction manuals or explicit training for new coders. Inspired by this human judgment, our network was exposed during the training and inference phases to multiple frames at once. We later report an ablation study showing that this approach significantly improved results. The neural network took as input a tensor $V\in R^{5x75x75x3}$ of five RGB (or grayscale) images of infant faces from consecutive alternating frames, and output a vector $U\in {\left[0,\;1\right]}^{3}$ representing the probability that the central frame (out of the five) will be “left,” “right,” or “away.” The cropped versions of faces were normalized channelwise by subtracting the mean and dividing by the standard deviation of the entire dataset, an established practice known for improving classification accuracy and enabling faster training (Simonyan & Zisserman, 2014). Training was performed using mini batches of 16 data points, as shown in the overview of network architecture in Figure 2. The categorical cross‐entropy loss was used with the Adam optimizer (Kingma & Ba, 2014), with an initial learning rate of 10^^−5^, which decreased over the epochs whenever validation loss stopped decreasing for three consecutive epochs. Training stopped when validation loss stopped decreasing for five consecutive epochs, namely when the inference process did not improve for five training steps. Note that during the inference phase (i.e., when making predictions on a new video), iCatcher needs five frames as input, but the face detector occasionally failed. If it failed for the target frame (i.e., the third frame out of the five), then it was labeled as “away,” since undetected faces are very likely associated with infants not looking at the screen. But if the failure occurred for one of the other frames, we replaced it with a black image, which signaled to the network that data were missing for that frame. We simulated this process during the training phase by randomly blacking out one image out of the five for each data point. This simulation step was observed to improve robustness to missing frames, while keeping general performance the same across the test set. Additionally, we randomly augmented the brightness of all the frames to better cope with the range of lighting conditions present in the dataset. This pre‐augmentation improved the f1‐score across all experiments by approximately 1% (see Results for description of the f1‐score metric). Lastly, we weighted the cost of misclassifying data points with a 1:0.66:0.66 ratio with respect to their classes (“away,” “left,” and “right,” respectively). This was motivated by the decreased number of samples in our dataset belonging to the “away” class, and the tendency of human annotators to have lower accuracy when coding “away,” which implies it is harder to label.

We performed comparisons of iCatcher to OpenFace (Baltrusaitis et al., 2018) and Real‐Time Eye Gaze in Natural Environments (RT‐GENE, Fischer et al., 2018). OpenFace was selected due to its established reputation in aiding facial behavior analysis, though its eye gaze tracking submodule (Wood et al., 2015) was shown to be less accurate than more recent solutions such as RT‐GENE using various metrics on multiple datasets (see Fischer et al., 2018 for more details). Both solutions are designed to provide a continuous eye gaze direction as output (instead of the desired 3‐class classification), but because they have shown robust success in predicting a continuous angular gaze vector from images, it was reasonable to assume they would succeed in our relatively simpler discrete problem space too. We designed several variations of these eye‐gaze estimation networks to fit our task. OpenFace was used as a black‐box feature extractor on our dataset (i.e., we extracted eye gaze features and information about facial position and rotation in an offline manner). We trained a fully connected neural network to classify these features into the discrete set of directions (“away,” “left,” and “right”). For RT‐GENE, since the trained models and weights were readily available, we further experimented by adjusting their solution to allow multiframe input and incorporating the face crop location along the horizontal axis as extra information. That is, it was concatenated as a deep feature into the network. We collectively refer to these setups and combinations of adjustments as the *RT‐GENE‐Like* setup, and the results reported below (Table 1), all indicate the best score achieved by any one of them.

**TABLE 1:** Weighted f1‐scores for eight videos in the test set

Method	Avg score (min–max)
Left versus Right versus Away	
Human annotators	96.7 (93.6–99.1)%
OpenFace	50.8 (34.7–65.1)%
RT‐GENE‐like	85.9 (78.3–92.1)%
iCatcher (single‐frame, with off‐the‐shelf face extraction)	85.8 (71.5–93.2)%
iCatcher (single‐frame)	85.9 (76.9–93.3)%
iCatcher (multi‐frame, with off‐the‐shelf face extraction)	89.8 (86.2–94.1)%
iCatcher (multi‐frame)	90.4 (84.7–95.0)%

Left versus Right only	
Human annotators	97.4 (94.4–99.4)%
iCatcher (multi‐frame)	99.6 (98.0–99.9)%

*Note*: We compared the independent results from both automated and human coding to the original human annotators. We first evaluated performance with three possible responses (left, right, or away), and report results for each method, ending with our iCatcher multiframe solution. We then evaluated performance with two possible responses (left or right).

### 2.3 Post processing

A very desirable property of our output is that it complies with certain constraints posed by the physical world. For example, because the video is recorded at a rate of 30 frames per second, it is not physically possible for an infant to shift the gaze direction from right to left (or left to right) in two consecutive frames, given that this behavioral transition takes much more time (Haith et al., 1993; Hood & Atkinson, 1993). Existing manual coding software pipelines directly encode these illegal transitions and do not permit them as annotation. One might assume that the network is expected to learn these rules implicitly when training; however, we process each data point (i.e., every quintuple of frames) individually at the inference phase, so the network is not actually exposed to these kinds of dependencies between predictions. To strictly enforce this, we passed the prediction of the neural network through a series of corrections such that the final output adhered to all constraints (e.g., switching immediately from “left” to “right” without “away” in between). We verified that this post‐processing step did not alter the results in a significant manner and allowed us to create output that can be easily analyzed using existing procedures for manually coded data.

## 3 RESULTS

We first report how human annotators perform relative to themselves and compare that with how iCatcher performs relative to humans for “left” versus “right” versus “away” (a 3‐class problem) and “left” versus “right” coding (a 2‐class problem). This was performed purely based on the labels produced by humans versus iCatcher for each valid frame (i.e., frames which were tagged by humans as valid and labeled). Second, we validated the results by using iCatcher to automatically code unseen data from a recent study that used manual coding (Potter & Lew‐Williams, 2022), and we show a successful replication of the original results.

### 3.1 General performance

**FIGURE 3:**
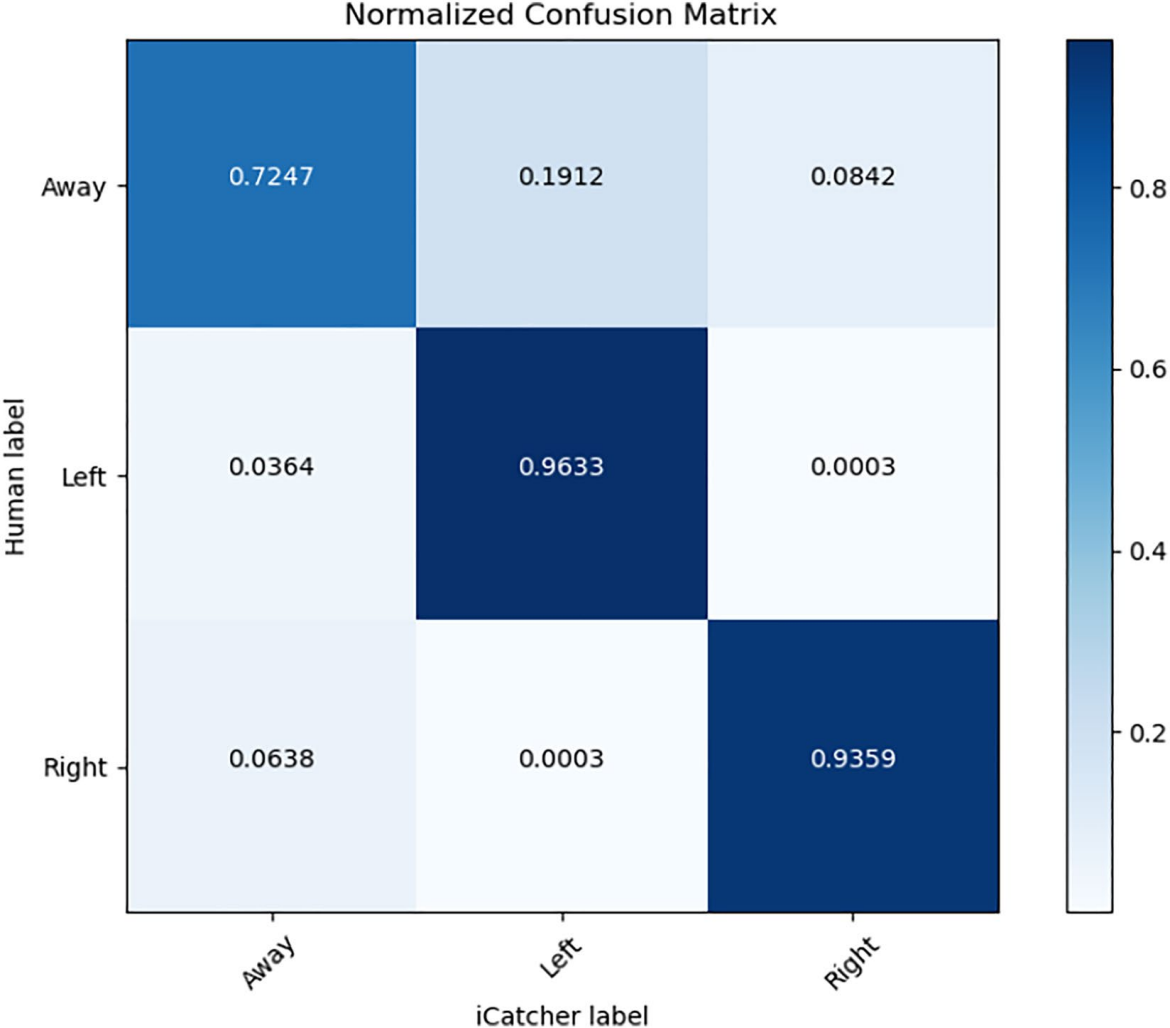
Normalized confusion matrix. *Note*. The confusion matrix is obtained by predicting on our test set using iCatcher's multiframe solution. The confusion between the away class and the other classes contributes the most to the model's error. Note the prediction score of left and right frames approximates that of human annotators (see Table 1)

To assess the model's success, we evaluated performance using an f‐1 score. We chose this metric, the harmonic mean of precision and recall averaged across eight test videos, because it is less biased than the straightforward accuracy percentage score and accounts for both false positives and false negatives. Performance scores are summarized in Table 1 and compared to results obtained using other methods. We found that all versions of iCatcher exceed the performance of OpenFace, and while the single‐frame RT‐GENE‐like setup performs comparably to the single‐frame versions of iCatcher, RT‐GENE requires significantly larger memory and computational resources and cannot match the performance of iCatcher's multiframe solution. Note that all videos were previously unseen by any of the methods and were the same videos used to establish intercoder reliability in the original investigation. Evaluation of the scores was performed in two ways: Firstly, each of the videos was fed into iCatcher and annotated automatically on the fly. This included using the face extractor and thus introduced new errors that arose from its accuracy and failures to detect faces. Configurations like these imitate expected performance in real time, and are referred to “with off‐the‐shelf face extraction” in Table 1. Second, to gain more insight into iCatcher's performance (and faithfully understand its errors), we processed the videos in an offline manner (extracting the faces) and manually verified them (This means the face detector's failures are discarded). iCatcher was then tested on this verified dataset, and our multiframe solution yielded a 90.4% f‐1 score with the original human annotation. Note that we included one video in which the child was sitting unusually off‐center relative to the screen; we see it as informative to include this extreme case even though it substantially lowered the mean and range of accuracy and may not be representative. Errors for this video were consistently larger using all automated methods (but not for humans). In the 3‐class problem, the confusion between the “away” class and other classes was observed to be the major reason for decreased performance using our method as can be seen in Figure 3. This is not surprising, since this was also the case with human annotators; right and left are rarely confused, but there may be a disagreement about whether the child is looking at a picture or off‐task.

Note that analyses of looking behavior tend to focus only on frames where the participant is fixating on one of two displayed images, making “right” and “left” classes more critical to label correctly. Encouragingly, our method indeed performed better for these classes (Figure 3). To strengthen this claim, we ran a separate setup where a similar network was trained only on “left” versus “right” (the 2‐class problem, with the last layer modified to fit a binary classification problem). This version reproduced the human annotations to an almost perfect degree (99.6%) on the aforementioned manually verified dataset (see “Left vs. Right Only” in Table 1). Note that this cannot be perfectly compared to human coders, because humans annotated frames using three options. However, it is very common in developmental science to only analyze left versus right, and the iCatcher's multiframe solution is much more successful in capturing this binary distinction than prior methods (e.g., Chouinard et al., 2019). In addition, while our dataset consisted of videos collected in a controlled lab setting, preliminary results with videos collected using the Lookit online platform (Scott & Schulz, 2017) suggest that the use of iCatcher will be generalizable. Even when presented with more variable videos from the Lookit platform, the iCatcher architecture was able to reliably classify the direction of infants' gaze (Cao et al., 2021). This extension suggests that the network will be a useful tool across different datasets and settings.

### 3.2 Comparisons to manually coded data

To further validate our multiframe, real‐time method, we reanalyzed all data from a recent study (Potter & Lew‐Williams, 2022) that had yielded a moderate but significant effect (main effect: *f = *0.37). This study was designed to test 24‐month‐old infants' ability to recognize high‐ and low‐frequency nouns (e.g., *horse* vs. *pony*) in typical versus atypical sentence frames (e.g., *Look at the horse* vs. *Examine the horse*). This created a 2 × 2 (target noun: high‐ vs. low‐frequency, sentence frame: typical vs. atypical) design, where we were able to compare children's accuracy across trial types. We followed common conventions in the field (Byers‐Heinlein et al., 2017; Fernald et al., 2008; Potter et al., 2019) and used identical procedures for cleaning and analyzing the data from both sources.

As in the original study (Potter & Lew‐Williams, 2022), we initially included videos from 39 participants who contributed up to 32 trials each (eight per condition). We then excluded trials and participants using the exact same methods and criteria. Trials were excluded if the participant was looking away at the beginning of our critical window of analysis (367–2000 ms after the onset of the target noun, 49 total frames), or if the child looked away for more than 15 consecutive frames during that window. Participants were excluded if they did not contribute useable data for at least two trials for each of the four trial types. In the original study, this resulted in the exclusion of five participants, and we ultimately analyzed 677 trials for 34 participants. In the current study, our final sample included 27 participants and we lost an additional 110 trials. We attribute this loss of data directly to the network's decreased performance on the “away” class, since it plays a vital role in the criterion set to select valid data. This increase in data loss is comparable to the 15% difference in data loss between manually coded data and data obtained using an automatic eye tracker report obtained by Venker et al. (2020).

**FIGURE 4:**
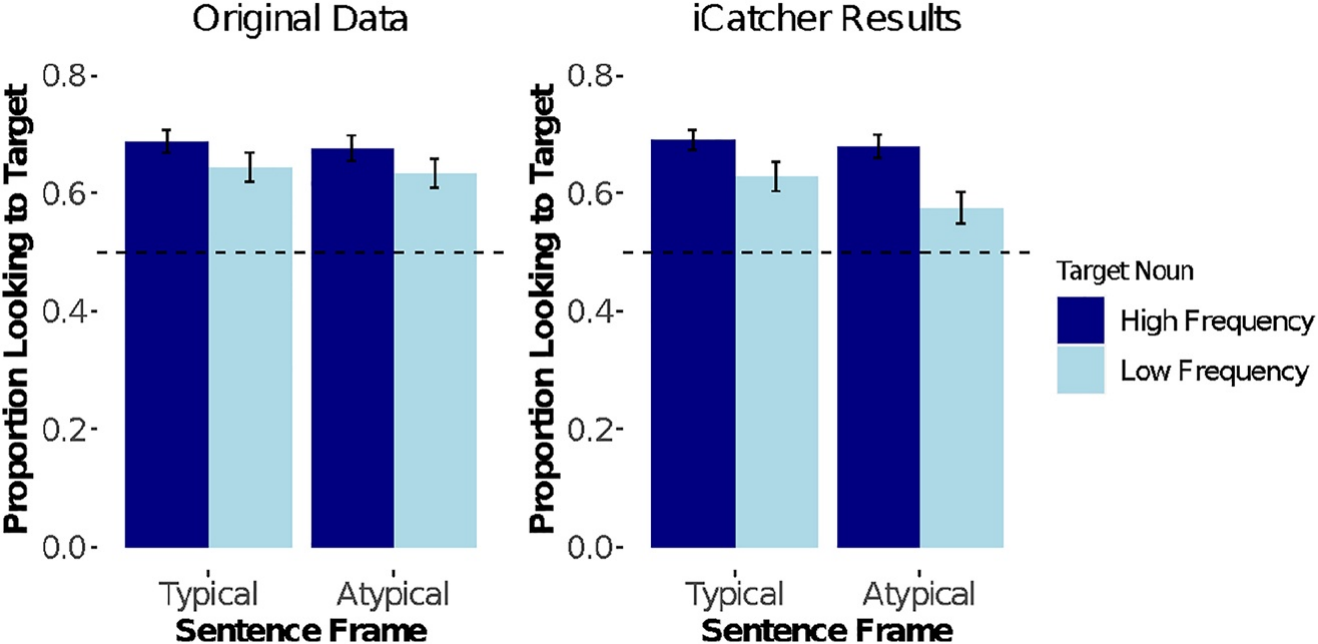
Comparison of human‐coded and network‐coded data. *Note*. Mean proportion of looking toward the target image during the target window, based on results from original human‐coded videos and those obtained using data from the network model. In both datasets, there is a significant main effect of Target Noun, and all individual conditions differ from chance, indicated by the dotted line. Error bars denote standard errors of the mean

But critically, despite using a slightly sparser dataset, results obtained using network‐coded data closely replicate the original patterns. We computed children's accuracy for each trial type. Accuracy was defined as the proportion of time children were looking toward the target image divided by the time spent looking at either the target or the distracter during the 49‐frame window of analysis. Note that frames in which the child was looking “away” are excluded from this calculation. We found that accuracy in all conditions was reliably above chance, *M* = 0.65, all *p* < 0.001, consistent with the original finding, *M* = 0.66, all *p* < 0.0001. We also again found a significant main effect of target noun, *F*(1, 26) = 5.99, *p* = 0.02, $\eta_{p}^{2}$ = 0.19, no significant effect of frame, *F*(1, 26) = 0.32, *p* = 0.57, and no significant interaction, *F*(1, 26) = 0.64, *p* = 0.43. These results are highly comparable to the original pattern, main effect of target noun: *F*(1, 33) = 4.36, *p* = .04, $\eta_{p}^{2}$ = 0.12; Frame : F(1, 33) = 0.25, *p* = 0.62; Interaction: *F*(1, 33) = 0.001, *p* = 0.97, see Figure 4. Thus, data coded by humans and by iCatcher yield the same interpretation.

## 4 DISCUSSION

Using an approach derived from state‐of‐the‐art methods in computer vision, our iCatcher network presents a new and realistic alternative for estimating the real‐time location of infants' eye gaze that does not require expensive equipment or too much human labor. We have demonstrated that this network can approximate human coders' accuracy in differentiating looks to the right versus left side of the screen. Moreover, in addition to per‐frame accuracy, we have shown that downstream use of data annotated using iCatcher yields the same pattern of results and psychological interpretation as those obtained using time‐intensive manual coding. We suggest that our approach offers a powerful tool for future developmental research that involves binary measures of looking behavior by providing high precision while requiring significantly smaller investment of time and money compared to existing techniques for coding eye movements.

One advantage of iCatcher is that it does not require any calibration of participant eye gaze, which is important for its utility in developmental science given that infants sometimes lose interest during the calibration or—even worse—partway through an experiment. Moreover, in several other available automated methods, participants must click on the location of a screen where they are looking, as in Papoutsaki et al. (2016); infants and young children are physically unable to perform this task. We also take advantage of real behavioral constraints. Infants typically cannot disengage attention or move their eyes in less than 200 ms (Haith et al., 1993; Hood & Atkinson, 1993). This fact was used in the design of iCatcher to process five alternating frames at the same time, which enhances the ability to “catch’ the trajectory of the eye movements across time. In manual coding, it is in fact common practice to use temporal information across frames to gain deeper understanding of data and thus achieve higher accuracy; that is, human annotators often backtrack a few frames (or more) to make corrections and best guesses. iCatcher imitates and automates this process by processing multiple frames at once. This feature proved crucial in surpassing previous fully automatic appearance‐based methods posed with the same task (OpenFace, RT‐GENE‐like, see Table 1). We attribute the weaker results obtained using OpenFace to the fact that it was used as a black box (i.e., it could not be retrained or fine‐tuned on our dataset) and was originally trained on adults who exhibit very different facial features than infants, thereby making it less useful for developmental research. The RT‐GENE‐like setup performed similarly to single‐frame versions of iCatcher, but it was not designed to deal with video streams or to capture temporal information in the same way as the multiframe version of iCatcher. Moreover, RT‐GENE requires significantly more memory and computational power, making it a less viable tool for many researchers.

In addition to outperforming other appearance‐based solutions, iCatcher exceeds prior attempts to classify infants' looking behavior (e.g., Chouinard et al., 2019). What is particularly promising about these results is that we not only achieve high accuracy in determining infants' gaze on a given frame, but also globally reproduce the results of a real study. Importantly, this did not have to be the case. The only other direct comparison between manual and automated methods of which we are aware (Venker et al., 2020) showed that even the relatively small amount of data loss produced by an automatic eye tracker could be enough to eliminate a significant effect. In contrast, we fully reproduced all significant (and nonsignificant) comparisons reported by Potter and Lew‐Williams (2022). This successful replication supports the likelihood that iCatcher can be used as a real and reliable tool in infant research.

**FIGURE 5:**
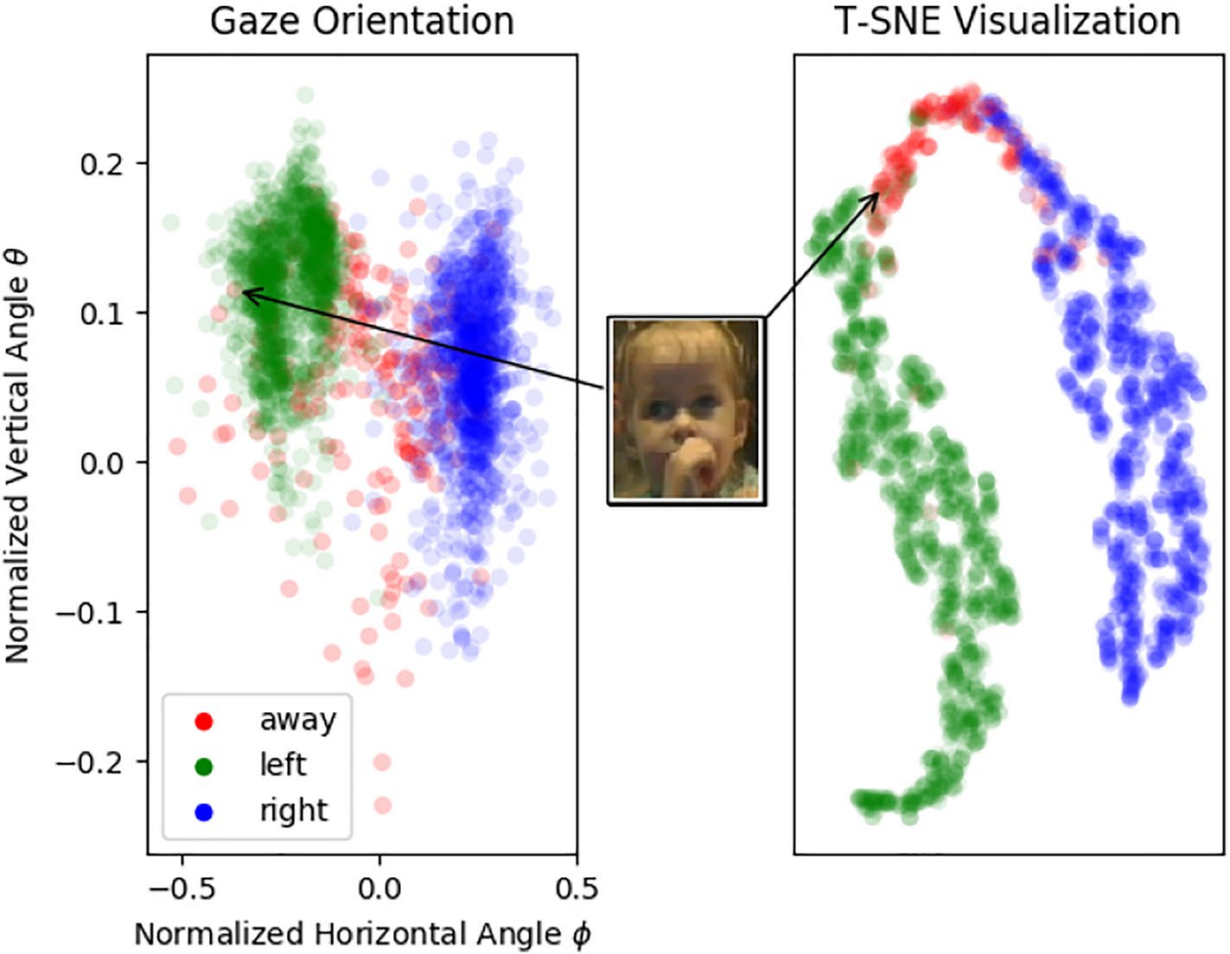
Incorporation of the horizontal shift of the face from the center of frame into the RT‐GENE‐like architecture. *Note*. Left: viewing angels as extracted by a “vanilla” version of RT‐GENE for held‐out video frames. Each frame is colored according to its manual coding. Right: the same frames, obtained at the output of the first fully connected layer within our version of the network (where the normalized horizontal shift was concatenated to the deep features), projected to 2D using T‐SNE (van der Maaten & Hinton, 2008). Notice how the incorporation of this feature enables the network to map the frames to more distinguishable clusters, as demonstrated by the pointed‐out “away” frame in both graphs

iCatcher does entail some disadvantages. The problem of estimating the discrete eye‐gaze direction using an appearance‐based method is an ill‐posed problem. Cameras, camera angles, and lighting conditions at different data collection sites can introduce great variability in video quality, which raises two issues. First, while two cropped face images might appear the same to the program, they could reflect a different actual gaze direction if the infant is located at the edge versus the center of the video. We believe many of the errors our network produces are due to these sorts of noisy states, all of which are related to the physical properties of the room in which the experiment is taking place, from which a purely appearance‐based model might not be able to learn or generalize. This was indeed the case for one of the videos used for testing, where the child was seated on the extreme left of the screen and all scores were particularly low. To solve this, it is likely that incorporating specific prior information about the room into the network can help with eliminating the problem (e.g., camera location, distance to screen, horizontal location of subject relative to screen, and size of screen). To support this claim, we inserted such prior information to the RT‐GENE‐like setup, where the horizontal locations of infants' faces were used as part of the input to the network, which enhanced the ability to classify frames dramatically. See Figure 5 for more information.

A second important limitation is that iCatcher is adapted to one set of laboratory conditions, as all training videos were recorded in the same room using the same camera and screen configuration. These factors make the solution less applicable for new locations. There are two ways to maximize what iCatcher has to offer. First, it would be advantageous to create a unified model that is trained on a larger, more diverse dataset that includes different participant groups, cameras, screen arrangements, and lighting conditions. This dataset would be a valuable resource, but it would also pose unique challenges in terms of organizing a large collaborative effort and obtaining broad consent to share participant videos across labs. A related approach would be to ask new users of iCatcher to create a small representative dataset from their own experimental setup (coded by humans) to be used for fine tuning the model in combination with our pretrained network. The product could then be shared, such that labs with similar setups might be able to use iCatcher with immediate success. In particular, we expect that training (or at least fine tuning) iCatcher on higher‐resolution videos would lead to rapid improvement in the network's performance, because higher quality videos include more intricate visual details and may enable higher precision in determining gaze direction. Testing this will require other annotated datasets, as in efforts by Cao et al. (2021), who made valuable adjustments to iCatcher that yielded increasingly reliable results. In our experience, training time for iCatcher takes less than a day (on a machine equipped with a strong GPU), and requires a dataset on the order of tens of thousands of data points (with each data point in iCatcher consisting of five frames).

Looking ahead, we suggest that iCatcher not only reduces the labor involved in transforming videos into data that can be readily analyzed, but can also be used to develop new paradigms that take advantage of both its speed during inference (25 frames per second) and precision. For instance, because data can be classified in real time with less than a second delay, it would be highly feasible to incorporate real‐time contingency into the design of the study. For example, trials in which the infant was inattentive could be repeated, providing a more complete dataset for each participant, or infants could be presented with harder or easier trials depending on their performance earlier in the task. Another possibility is that our approach could be enhanced over time to produce more continuous gaze directions (i.e., horizontal and vertical angles), which could open the possibility for studies that require more subtle and dynamic stimuli than can currently be reliably judged by humans, which would be more akin to the existing capabilities of automatic eye trackers. Perhaps most notably, given current directions in research, iCatcher shows high potential for integration with online platforms, which could allow us to collect data from many different populations and locations on a scale that is currently impractical for most developmental scientists.

## CONFLICT OF INTEREST

The authors declare no conflicts of interest with regard to the funding source for this study.

## References

[infa12468-bib-0001] Aslin, R. N. (2007). What's in a look? Developmental Science, 10(1), 48–53. 17181699 10.1111/J.1467-7687.2007.00563.X PMC2493049

[infa12468-bib-0002] Aslin, R. N. (2012). Infant eyes: A window on cognitive development. Infancy, 17(1), 126–140. 22267956 10.1111/j.1532-7078.2011.00097.x PMC3259733

[infa12468-bib-0003] Baltrusaitis, T. , Zadeh, A. , Lim, Y. C. , & Morency, L. (2018). OpenFace 2.0: Facial behavior analysis toolkit. In 13th IEEE International Conference on Automatic Face & Gesture Recognition (FG 2018), 2018, (pp. 59–66).

[infa12468-bib-0004] Bergelson, E. , & Swingley, D. (2012). At 6–9 months, human infants know the meanings of many common nouns. Proceedings of the National Academy of Sciences, 109(9), 3253–3258. 10.1073/pnas.1113380109 PMC3295309 22331874

[infa12468-bib-0005] Bradski, G. (2000). The OpenCV library. Dr. Dobb's Journal of Software Tools.

[infa12468-bib-0006] Byers‐Heinlein, K. , Morin‐Lessard, E. , & Lew‐Williams, C. (2017). Bilingual infants control their languages as they listen. Proceedings of the National Academy of Sciences, 114(34), 9032–9037. 10.1073/pnas.1703220114 PMC5576790 28784802

[infa12468-bib-0007] Cao, P. , Tan, X. , Scott, K. , & Liu, S. (2021). iCatcher+: Robust and automatic gaze classification of infant webcam videos. PsyArXiv. 10.31234/osf.io/s8c4m

[infa12468-bib-0008] Chouinard, B. , Scott, K. , & Cusack, R. (2019). Using automatic face analysis to score infant behaviour from video collected online. Infant Behavior and Development, 54, 1–12. 30508782 10.1016/j.infbeh.2018.11.004

[infa12468-bib-0009] Eberhard, K. M. , Spivey‐Knowlton, M. J. , Sedivy, J. C. , & Tanenhaus, M. K. (1995). Eye movements as a window into real‐time spoken language comprehension in natural contexts. Journal of Psycholinguistic Research, 24(6), 409–436. 8531168 10.1007/BF02143160

[infa12468-bib-0010] Fagan, J. F., III (1974). Infant recognition memory: The effects of length of familiarization and type of discrimination task. Child Development, 45, 351–356. 4837713 10.1111/j.1467-8624.1974.tb00603.x

[infa12468-bib-0011] Fernald, A. , Pinto, J. P. , Swingley, D. , Weinbergy, A. , & McRoberts, G. W. (1998). Rapid gains in speed of verbal processing by infants in the 2nd year. Psychological Science, 9(3), 228–231.

[infa12468-bib-0012] Fernald, A. , Zangl, R. , Portillo, A. L. , & Marchman, V. A. (2008). Looking while listening: Using eye movements to monitor spoken language. Developmental psycholinguistics: On‐line methods in children's language processing, 44, 97–135.

[infa12468-bib-0013] Fischer, T. , Jin Chang, H. , & Demiris, Y. (2018). Rt‐gene: Real‐time eye gaze estimation in natural environments. In Proceedings of the European Conference on Computer Vision (ECCV).

[infa12468-bib-0014] Golinkoff, R. M. , Hirsh‐Pasek, K. , Cauley, K. M. , & Gordon, L. (1987). The eyes have it: Lexical and syntactic comprehension in a new paradigm. Journal of Child Language, 14(1), 23–45. 3558524 10.1017/s030500090001271x

[infa12468-bib-0015] Gredebäck, G. , Johnson, S. , & von Hofsten, C. (2009). Eye tracking in infancy research. Developmental Neuropsychology, 35(1), 1–19. 10.1080/87565640903325758 20390589

[infa12468-bib-0016] Haith, M. M. , Wentworth, N. , & Canfield, R. L. (1993). The formation of expectations in early infancy. Advances in Infancy Research.

[infa12468-bib-0017] Hood, B. M. , & Atkinson, J. (1993). Disengaging visual attention in the infant and adult. Infant Behavior and Development, 16(4), 405–422.

[infa12468-bib-0018] Kingma, D. P. , & Ba, J. (2014). Adam: A method for stochastic optimization.

[infa12468-bib-0019] Kinzler, K. D. , Dupoux, E. , & Spelke, E. S. (2007). The native language of social cognition. Proceedings of the National Academy of Sciences, 104(30), 12577–12580. 10.1073/pnas.0705345104 PMC1941511 17640881

[infa12468-bib-0020] Krafka, K. , Khosla, A. , Kellnhofer, P. , Kannan, H. , Bhandarkar, S. , Matusik, W. , & Torralba, A. (2016). Eye tracking for everyone. In 2016 IEEE Conference on Computer Vision and Pattern Recognition (CVPR). 10.1109/CVPR.2016.239

[infa12468-bib-0021] Krizhevsky, A. , Nair, V. , & Hinton, G. (2020). CIFAR‐10 (Canadian Institute for Advanced Research). http://www.cs.toronto.edu/∼kriz/cifar.html

[infa12468-bib-0022] Lew‐Williams, C. , & Fernald, A. (2007). Young children learning Spanish make rapid use of grammatical gender in spoken word recognition. Psychological Science, 18(3), 193–198. 17444909 10.1111/j.1467-9280.2007.01871.x PMC3206966

[infa12468-bib-0023] Maccoby, E. (2019). Eleanor Maccoby: An abridged memoir. Annual Review of Developmental Psychology, 1, 1–20.

[infa12468-bib-0024] Naigles, L. R. , & Tovar, A. T. (2012). Portable intermodal preferential looking (IPL): Investigating language comprehension in typically developing toddlers and young children with autism. Journal of Visualized Experiments, 70, e4331. 10.3791/4331 PMC3570064 23271456

[infa12468-bib-0025] Oakes, L. M. (2010). Editorial comment: Infancy guidelines for publishing eye‐tracking data. Infancy, 1(15), 1–5. 10.1111/j.1532-7078.2010.00030.x 32693459

[infa12468-bib-0026] Papoutsaki, A. , Sangkloy, P. , Laskey, J. , Daskalova, N. , Huang, J. , & Hays, J. (2016). Webgazer: Scalable webcam eye tracking using user interactions. In Proceedings of the 25th International Joint Conference on Artificial Intelligence (IJCAIi). AAAI.

[infa12468-bib-0027] Potter, C. E. , Fourakis, E. , Morin‐Lessard, E. , Byers‐Heinlein, K. , & Lew‐Williams, C. (2019). Bilingual toddlers' comprehension of mixed sentences is asymmetrical across their two languages. Developmental Science, 22(4), e12794. 30582256 10.1111/desc.12794 PMC6570532

[infa12468-bib-0028] Potter, C. E. , & Lew‐Williams, C. (2022). Frequent vs. infrequent words shape toddlers’ real‐time sentence processing. PsyArXiv. 10.31234/osf.io/mertp PMC10764636 37401467

[infa12468-bib-0029] Ross‐Sheehy, S. , Oakes, L. M. , & Luck, S. J. (2003). The development of visual short‐term memory capacity in infants. Child Development, 74(6), 1807–1822. 14669897 10.1046/j.1467-8624.2003.00639.x

[infa12468-bib-0030] Russakovsky, O. , Deng, J. , Su, H. , Krause, J. , Satheesh, S. , Ma, S. , Huang, Z. , Karpathy, A. , Khosla, A. , Bernstein, M. , Berg, A. C. , & Fei‐Fei, L. (2015). ImageNet large scale visual recognition challenge. International Journal of Computer Vision, 115(3), 211–252. 10.1007/s11263-015-0816-y

[infa12468-bib-0031] Scott, K. , & Schulz, L. (2017). Lookit (part 1): A new online platform for developmental research. Open Mind, 1(1), 4–14.

[infa12468-bib-0032] Simonyan, K. , & Zisserman, A. (2014). Very deep convolutional networks for large‐scale image recognition. arXiv 1409.1556.

[infa12468-bib-0033] Trueswell, J. C. (2008). Using eye movements as a developmental measure within psycholinguistics. Language Acquisition and Language Disorders, 44, 73–96.

[infa12468-bib-0034] Trueswell, J. C. , & Gleitman, L. (2004). Children's eye movements during listening: Developmental evidence for a constraint‐based theory of sentence processing. The interface of language, vision, and action: Eye movements and the visual world, 319–346.

[infa12468-bib-0035] van der Maaten, L. , & Hinton, G. (2008). Visualizing data using t‐SNE. Journal of Machine Learning Research, 9, 2579–2605. http://www.jmlr.org/papers/v9/vandermaaten08a.html

[infa12468-bib-0036] Venker, C. E. , Pomper, R. , Mahr, T. , Edwards, J. , Saffran, J. , & Ellis Weismer, S. (2020). Comparing automatic eye tracking and manual gaze coding methods in young children with autism spectrum disorder. Autism Research, 13(2), 271–283. 31622050 10.1002/aur.2225 PMC7359753

[infa12468-bib-0037] Wass, S. V. , Forssman, L. , & Leppänen, J. (2014). Robustness and precision: How data quality may influence key dependent variables in infant eye‐tracker analyses. Infancy, 19(5), 427–460.

[infa12468-bib-0038] Wood, E. , Baltruaitis, T. , Zhang, X. , Sugano, Y. , Robinson, P. , & Bulling, A. (2015). Rendering of eyes for eye‐shape registration and gaze estimation. In 2015 IEEE International Conference on Computer Vision (ICCV), (pp. 3756–3764).

[infa12468-bib-0039] Zhang, X. , Sugano, Y. , Fritz, M. , & Bulling, A. (2015). Appearance‐based gaze estimation in the wild. In Proceedings of the IEEE Conference on Computer Vision and Pattern Recognition (CVPR).

[infa12468-bib-0040] Zieliński, P. (2020). Opengazer: Open‐source gaze tracker for ordinary webcams (software). Samsung and the Gatsby Charitable Foundation. http://www.inference.org.uk/opengazer

